# Study on Differences between Patients with Physiological and Psychological Diseases in Online Health Communities: Topic Analysis and Sentiment Analysis

**DOI:** 10.3390/ijerph17051508

**Published:** 2020-02-26

**Authors:** Jingfang Liu, Jun Kong, Xin Zhang

**Affiliations:** School of Management, Shanghai University, Shanghai 201800, China; jingfangliu2014@hotmail.com (J.L.); 513651789@i.shu.edu.cn (X.Z.)

**Keywords:** disease difference, online health community, topic modeling, sentiment analysis

## Abstract

The development of online social platforms has promoted the improvement of online health communities (OHCs). However, OHCs often ignore differences in user discussions caused by the characteristics of diseases. The purpose of this research was to study differences in the topics and emotions of patients with physiological and psychological diseases by mining the text that they posted in OHCs as well as to discuss how to satisfy these differences. The data came from Baidu Post Bar, the world’s biggest Chinese forum. We collected 50,230 posts from heart disease, hypertension, depression and obsessive-compulsive bars. Then, we used topic modeling and sentiment analysis techniques on these posts. The results indicate that there are significant differences in the preferences of discussion and emotion between patients with physiological and psychological diseases. First, people with physiological diseases are more likely to discuss treatment of their illness, while people with psychological diseases are more likely to discuss feelings and living conditions. Second, psychological disease patients’ posts included more extreme and negative emotions than those of physiological disease patients. These results are helpful for society to provide accurate medical assistance based on disease type to different patients, perfecting the national medical service system.

## 1. Introduction

### 1.1. Background

With the booming development of online communities, the Internet provides a variety of online social platforms for public participation, where users can actively express their opinions and communicate with others in a convenient and timely manner [1]. Therefore, massive amounts of valuable user-generated content (UGC) can be used for academic research. By participating in online social platforms, users may not only obtain information from online communities unilaterally, they may also get acquainted with like-minded friends on different platforms and online communities to interact with them, share experiences and provide social support [2,3]. In the field of healthcare, the booming development of online social platforms has promoted the improvement of relevant platforms themed on healthcare and has given rise to the term “online health community” (OHC). Based on Internet technology, online health communities are a platform to discuss disease-related problems, share medical experiences, provide remote medical service and organize member activities [4,5,6,7].

The main participants of online health communities are generally patients, relatives or people with health management needs. According to a report released by a big data research institution in 2017, by the end of 2016, the user scale of China’s medical health market was nearly 300 million [8]. Dividing users according to the main community in which they participate, and then studying their behaviors, is conducive to deepening the understanding of this group, and understanding the behavioral differences among these groups is conducive to providing specific interventions or support behaviors [9,10]. Group classification of patients with chronic diseases is also beneficial to the study of the differences in the needs of different patients. It is the simplest and most reasonable to divide patients into groups based on physiological and psychological diseases. Understanding the demands of these diverse groups would be beneficial to online health community managers for improving user activity and increasing user stickiness. In addition, due to the large number of people living with chronic diseases in the world, understanding the medical needs and solving the health problems of patients with chronic diseases through online health communities are important ways to help with the development of health measures.

In general, user participation in online health community communication takes the form of questions: One asks a question and another responds, establishing social relations and providing social support based on their own opinions. The question and the corresponding answer follow a specific topic for discussion [11]. Topic modeling and sentiment analysis are often used to analyze UGC in online communities in order to uncover topics and emotions hidden in user discussion texts [12,13,14,15]. To explore the differences in interest topics between patients with physiological and psychological diseases, we used the topic-modeling methodology to mine the discussion topics of patients. Due to the subjectivity of online health community texts, differences in users’ emotions were also the focus of our attention. Sentiment analysis can help us understand the emotional differences of patients with different diseases. Combined with the results of topic modeling, we can deeply understand the unmet potential needs of patients.

The structure of this article is as follows. The first part introduces related research on topic and sentiment analysis of online health community content. Part two introduces the research framework and methods used in this study, including the acquisition of experimental samples, the data cleaning process and the algorithms. Then, in the third part, we explain the process and results of topic modeling and sentiment analysis. The research contribution and future directions are summarized in the fourth part.

### 1.2. Related Work

In terms of topic modeling in online health communities, many scholars have made great progress in the development of topic discovery in online health communities. Their work has examined the topics of text posted by patients with various chronic diseases as well as with the exchange of information and social support embodied in these topics.

As early as 2007, scholars Buchanan and Coulson used online questionnaires to explore the prominent topics of patients with anxiety or phobia in the online community: no longer feeling lonely, sharing experiences, providing and receiving information and gaining power [16]. Van Uden-Kraan et al. conducted a topic analysis of the text of patients with breast cancer, arthritis and fibromyalgia and found that the subjects associated with the diseases included daily living conditions and therapeutic drugs [17]. Similarly, in a study by Chen, he used the K-means method to cluster the text from three online health communities (breast cancer, type I diabetes and fibromyalgia) and discovered the same topics in each: patient experience, treatment, medication and physical management [18]. Perrone et al., from the perspective of social support theory, distinguished social support categories and deduced the topic of the text of a rare diabetic patient. They found that the most popular topic of the community concerned seeking or providing social support, followed by psychological and emotional support [19]. Using the same method described in this paper, Liu et al. also explored the user-generated content of online health communities. Using the Latent Dirichlet Allocation (LDA) method to explore topics in an online diabetes community in China, they found that the six topics discussed by patients included friends’ and doctors’ advice, exercise and diet, hypoglycemia and diet, diabetes research, glycation effects and risks as well as the use of medical instruments [9]. However, there are two main differences between our study. First, because Liu’s research used a specific disease as its subject, the text from the diabetes community cannot be compared with that of another community. In our study, text was acquired from different disease zones of the same forum. Thus, the cross-contrast of diseases can be achieved while differences in text caused by different platforms can be minimized. Second, Liu’s research divided patients according to gender, neglecting differences attributable to diseases characteristics. This article distinguished between physiological and psychological diseases, attaching importance to the disease category and their characteristics, which is more conducive to building specialized online communities for patients with different types of diseases.

Sentiment analysis is the process of analyzing, summarizing and inferring the emotion in text, and it aims to discover the speaker’s attitude toward a specific topic [20]. Sentiment analysis is often used in comments on goods and services [21], as well as social networks such as Twitter and Weibo [22,23]. Through sentiment analysis, it has been found that there are many methods to evaluate the service provided by doctors and to detect patients’ attitudes in online health communities. For instance, Salathé explored Twitter users’ attitudes toward a new vaccine and found that they were either positive or negative, with little neutrality [24]. Zhang et al. analyzed patients on the breast cancer forum and found that their emotions were the most negative when they joined the community, but that gradually increased. After long-term participation in the community, their emotions gradually became positive and stable, and their role gradually changed to being the provider of information and/or emotional support [25]. This paper adopts the method of analyzing emotions based on the sentiment dictionary. The purpose of this method is to judge whether the text emotion is positive, negative or neutral through the sentiment dictionary and its calculation rules.

Sentiment analysis is considered to be of particular significance to patients with psychological diseases. The emotions expressed in text may be considered to be reflective of the discussion atmosphere of a topic. Even if the subject is the same, the atmosphere of discussion of psychological disease patients may be completely different from that of physiological disease patients [26]. By analyzing the text published by users through social media, early psychological patients are likely to be found. Ortigosa et al. demonstrated a new emotion detection scheme for Facebook that automatically extracts text of emotional extremes from user-published texts, such as positive, neutral and negative emotions, to identify huge emotional differences among users [27]. Islam et al. used several machine-learning methods to judge depression, proving that Facebook text can be used to identify patients with depression [28]. Therefore, sentiment analysis of patients with physiological and psychological diseases could be helpful for identifying their differences with regard to emotional extremes in the community, and the degree of satisfaction among the two groups of patients toward the topics discussed can be understood by comparing these differences.

### 1.3. Research Gap

Currently, research on online health community user-generated content mostly targets single diseases or multiple diseases, but rarely does it analyze differences in the text of patients with different types of diseases. Therefore, we analyzed differences in text posted by patients with physiological and psychological diseases, using the methods of topic modeling and sentiment analysis to analyze the different interests and emotions expressed by the two groups of patients, discussing the potential needs of the patients based on the results of topic modeling and sentiment analysis.

## 2. Materials and Methods

### 2.1. Research Flowchart

**Figure 1:**
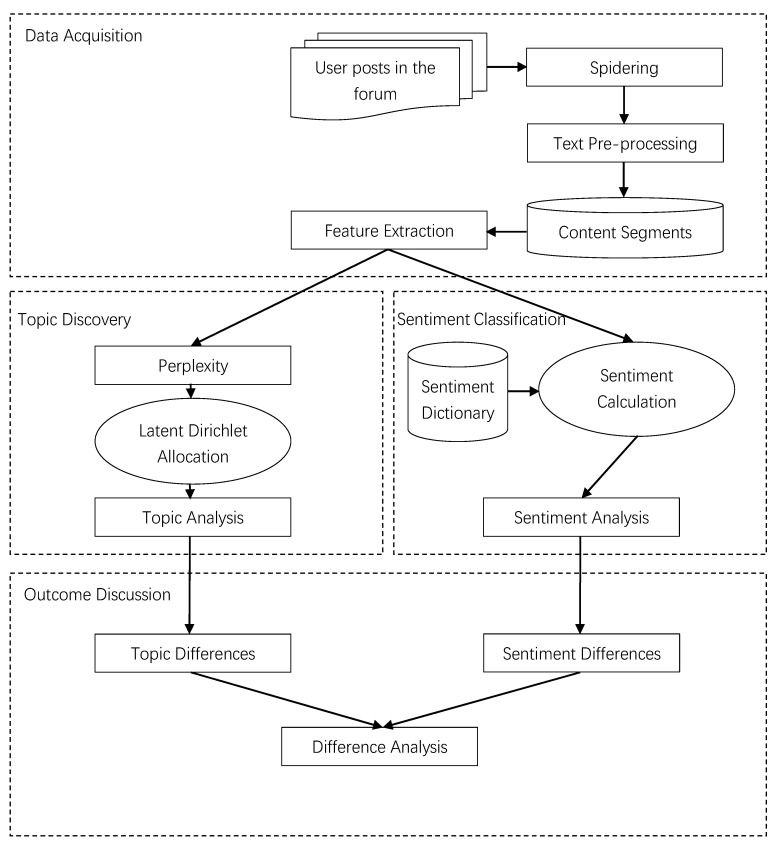
Research framework.

The proposed framework for this study is shown in Figure 1. The research is divided into three main parts: The first part concerns raw data acquisition and data preprocessing. The second part consists of two main procedures: topic modeling and sentiment analysis. The last part explores differences between patients with physiological and psychological diseases based on the results of the topic modeling and sentiment analysis, and it discusses the differences further.

### 2.2. Data

The dataset was collected from Baidu Post Bar, the world’s largest Chinese community, which allows users to search for different keywords to enter an online community associated with those keywords. In this community, users can publish or reply to posts freely, participate in post discussions, get information and make friends by browsing other posts. We applied the Python program to obtain data from user posts in the Baidu heart disease, hypertension, depression and obsessive-compulsive disorder (OCD) bars. To ensure that the text was written by the patient, a human screening was performed to improve the veracity of the dataset. We employed 4 graduate students as data proofreaders to screen 15,000 texts each, and removed the text posted by patient relatives rather than themselves, finally obtaining a raw dataset containing 50,230 texts. Our dataset is shown in Table 1.

### 2.3. Preprocessing

**Figure 2:**
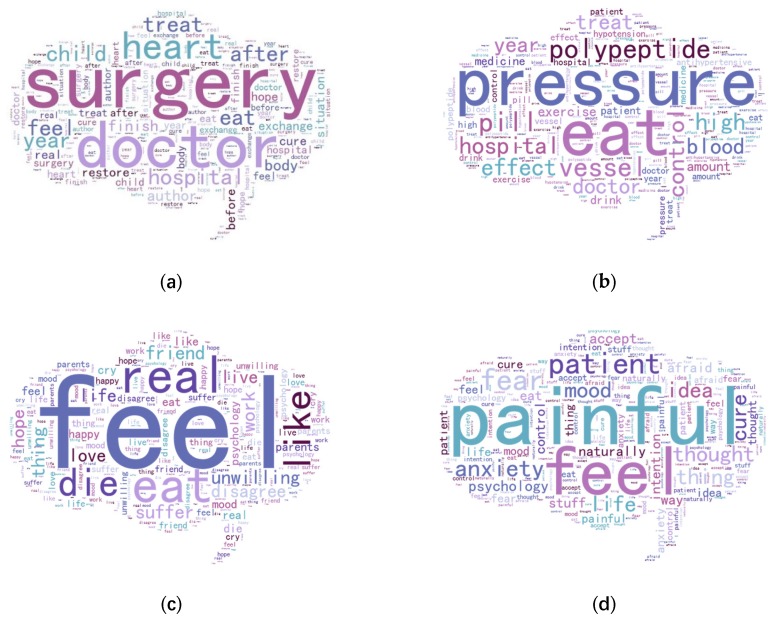
Word clouds in each community: (**a**) The word cloud from the heart disease online health community (OHC); (**b**) the word cloud from the hypertension OHC; (**c**) the word cloud from the depression OHC; and (**d**) the word cloud from the OCD OHC.

To better understand the text, we segmented the original corpus and removed punctuation marks that were not related to the content of the text. In addition, we addressed some text or words in a particular way. First, because the word segmentation results included punctuation marks, when the word segmentation results were less than 20, the substantive words may have been fewer, most likely only including exclamations or simple questions of off-topic text. Second, we removed text pertaining to location, number, web address and other irrelevant information from the text. Finally, we removed the words “heart disease”, “hypertension”, “depression” and “obsessive” from the text to avoid having many topic words directly associated with the diseases. After data preprocessing, a corpus of 17,891 documents comprising 509,250 words was obtained. The top 20 keywords for each community are shown in Figure 2a–d. As the figure shows, the most frequent words for the heart disease, hypertension, depression and OCD communities were surgery, pressure, feel and painful, respectively. Unsurprisingly, many of these words are associated with the characteristics of the disease itself.

In this paper, the TF–IDF algorithm was used to transform the text, and the feature words from the text were extracted according to the TF–IDF value [18], which can be used to extract or classify the topic words of articles. After calculating the TF–IDF values, we deleted words with TF–IDF values less than 0.10. These kinds of words usually appear in the corpus in large numbers, meaning that words not only reduce the accuracy of the subject division but also increase the calculation time. Finally, we generated a corpus with 17,891 posts comprising 44,227 feature words (including duplicate values).

### 2.4. Topic Modeling

To clarify the differences in discussion topics between patients with physiological and psychological diseases who participated in an online health community, we used the topic-generation model of LDA to model the text of patient discussions. The LDA model is a three-tier Bayesian topic-generation model proposed by Blei et al., which assumes that all documents in the corpus discuss several topics together and that each document has a different probability for each discussed topic [29]. The LDA model is widely used in text topic mining [30], text classification [31] and recommendation systems [32,33].

### 2.5. Topic Number Determination

The main problem with using the LDA topic-generation model is the determination of the number of topics. It is required to avoid omitting valuable topics and to ensure the interpretability of the topics. In our study, the optimal number of topics was determined using the perplexity value. The formula for calculating the perplexity value is as follows:

(1) $$perplexity\left(D\right)=exp\left|-\frac{\sum_{i=1}^{M}log(P\left(w_{i}\right)}{\sum_{i=1}^{M}N_{i}}\right|$$

where M is the number of texts tested in set D, $N_{i}$ is the length of the text $w_{i}\;$ and $P\left(w_{i}\right)$ is the probability of generating the text $w_{i}$ from the LDA model. The perplexity of the LDA model measures the stability of the LDA topic model based on model generalizability. It also ensures that the LDA model results in the predictability of new topics. A lower degree of perplexity indicates better model generalizability.

## 3. Results

### 3.1. Perplexity

**Figure 3:**
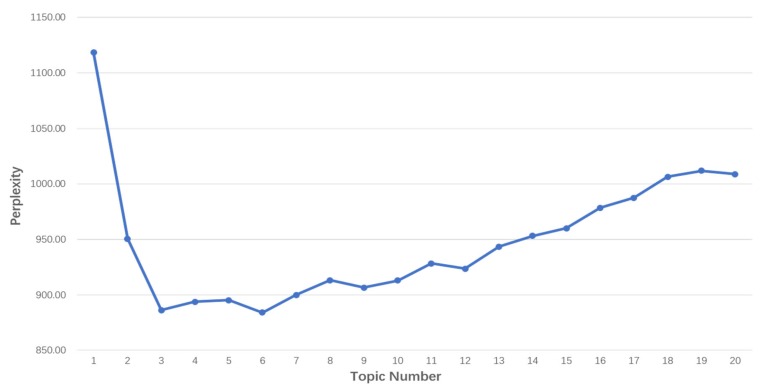
The perplexity change graph.

To determine the optimal number of topics, we adopted the method of calculating the perplexity in each number of topics. Based on the perplexity principle, a smaller value of perplexity reflects greater generalizability of the model [31]. Figure 3 shows a line graph of the perplexity, in which the horizontal axis represents the number of optional topics, and the vertical axis represents the perplexity value. 

As shown in Figure 3, we chose topic number in the interval N∈ [1,20], which ensured that valuable topics could be discovered and that each topic could be well-explained. The values of perplexity first decreased rapidly as the number of topics increased. It reached its lowest point at six topics and then continuously increased as the number of topics increased. Therefore, with the number of topic is six, the model was the most stable, and the intelligibility of the topic words was the best.

### 3.2. LDA Topic Model

The LDA topic model applied a Python program, and Gibbs sampling was used to estimate the model parameters during the modeling process. We performed 1500 iterations; after that, we acquired the most presentative words in each topic and summarized the top 10 words. Table 2 shows the top 10 topic words for the six topics that were ultimately generated.

Table 2 had given some clues that enabled us to explain and summarize the discussion text in each topic.

Topic 1 contains words describing feelings, such as “feel” and “thought”, as well as words describing emotions, such as “anxiety” and “painful”, which may be understood as patients describing feelings and ideas generated by external things.

Topic 2 includes words such as “accept”, “face” and “understand”. It can be observed that, under this topic, patients mainly describe their self-regulation when they face their diseases or the persuasion of others. 

Topic 3 includes the words “parents”, “friends” and “children”, which reflect the close social relationships of patients, as well as “work”, “life” and other key words that reflect social life. Therefore, it is obvious that patients mainly discussed their social environment in this topic, including mainly social relationships and living background.

Topic 4 includes key words such as “doctor”, “hospital” and other entities, as well as verbs such as “surgery” and “inspect”. Thus, this topic can be summarized as nondrug therapy, making it easy to distinguish from drug therapy.

Topic 5 also includes entity keywords such as “doctor” and “hospital”, but it differs from Topic 4 in that key words include “medicine”, “meal”, “effect” and other words that are highly relevant to drug therapy. Thus, this topic is summarized as “drug therapy”. Doctors usually advise patients to exercise when using drug therapy. Thus, the keyword “exercise” also appears under this topic.

The key words of Topic 6 mainly include the words “polypeptide”, “vessel” and pharmacological reaction words, such as “effect”, “ingredient”, and so on. Therefore, we inferred that this topic mostly consisted of disease knowledge and drug recommendations; that is, exchanges of professional knowledge.

To demonstrate that our explanation of each topic is specific enough and to enhance the understanding of these topics by clarifying more details of the differences between patients with physiological and psychological diseases, Table 3 provides examples of patient discussion text from four disease communities. The probability of each text that belongs to the topic is over 60 percent, which ensure the specificity of the text topic.

By observing the representative texts in Topic 1—the patients’ feelings and thoughts—we find that patients with physiological diseases were more likely to discuss their own thoughts or plans for action, while patients with psychological diseases were more likely to discuss how the diseases affected them. Both of the discussions reflect in this topic.

In the examples of Topic 2—patient self-regulation—we found that both physiological and psychological disease communities discussed their own self-regulation. This kind of self-regulation is mainly reflected in patients’ attitude adjustment and emotional control. In the forum, the topic of self-regulation is reflected in whether patients encourage others or comfort themselves.

After further observing characteristics of text in Topic 3—the social environment—we found that patients with physiological diseases were concerned with changes in their social environment for a period of time. In comparison, posts by patients with psychological diseases indicated that they pay more attention to the long-term social environment before and after the illness or to their main social relationships. We can identify this topic by the examples.

Topic 4—nondrug therapy. Physiological diseases can be treated directly with nondrug therapies, the text of the discussion on heart disease and hypertension is reflected in descriptions of surgery or laboratory tests, while psychological disease patients with depression or OCD are more discussed in the process of inspection and psychological interventions, which are related to the particularities of the disease itself.

By observing the posts assigned to Topic 5—drug therapy—we found that both patients with physiological and psychological diseases required certain drug therapies. Therefore, there is no significant difference in the text of these posts between the two disease communities.

We found that posts discussed by patients within Topic 6—professional knowledge exchange—involved a large number of drug recommendations in advertising texts or introducing professional knowledge. In contrast to the posts from the previous five topics, the words associated with Topic 6 were more specialized, and the text was not generated by normal community users. Therefore, although we did not analyze text differences under this topic, the existence of this topic shows patients’ need for professional medical and pharmacological knowledge. This certainly should be of great significance to the operators of online health communities.

**Figure 4:**
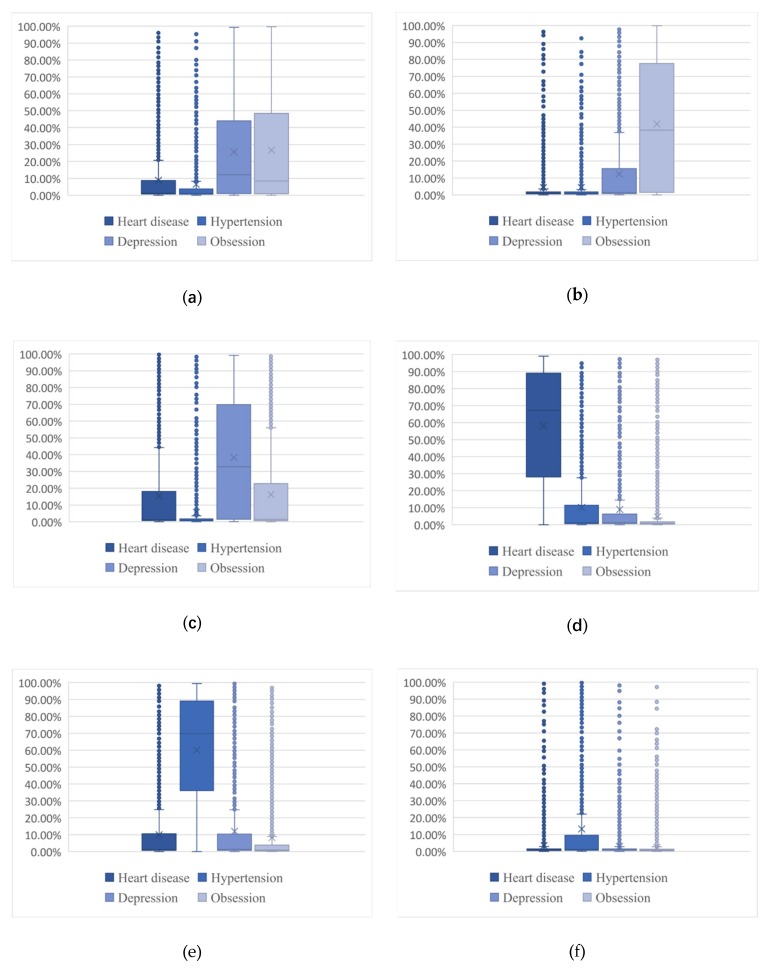
Text topic probability distribution: (**a**) The probability in Topic 1; (**b**) the probability in Topic 2; (**c**) the probability in Topic 3; (**d**) the probability in Topic 4; (**e**) the probability in Topic 5; and (**f**) the probability in Topic 6.

To assess the extent to which the topics may reflect patients’ discussion preferences, we plotted Figure 4a–f to analyze the probability distribution of each disease OHC for each topic. This step was also used to test the validity of the topic model to ensure that the topic model identified the discussion preferences of patients with different types of diseases. As a whole, the topics could clearly identify the differences in topics discussed by patients with different types of physiological and psychological diseases.

Figure 4a–f can clearly distinguish the discussion preference differences between the two kinds of patients. It can be seen in Figure 4 b that patients with obsessive-compulsive disorder were more likely to discuss Topic 2, patient self-regulation. In Figure 4c, patients with depression were more likely to discuss Topic 3, social environment. These topics focus on the expression of patients’ own emotions or the description of their living environment. Thus, patients with psychological diseases were more likely to focus on releasing their emotions and displaying their lives in an online health community. However, as shown in Figure 4d, patients with heart disease were more likely to discuss Topic 4, nondrug therapies. In Figure 4e, patients with hypertension were more likely to discuss drug therapies. We can conclude that patients with physiological diseases were more likely to be interested in the treatment of their diseases and were more likely to share their own or others’ treatment experiences. Finally, the discussions in Topic 1 can also illustrate the differences in topic preference between patients with physiological and psychological diseases. As shown in Figure 4a, patients with heart disease and hypertension participated in significantly less discussion pertaining to the topic of “feelings and thoughts” than did patients with depression and OCD; that is, patients with psychological diseases were more willing to express their thoughts, describe their feelings and express positive emotions that were beneficial to community activity.

### 3.3. Sentiment Analysis

We adopted the Boson NLP Chinese Sentiment Dictionary to analyze the text posted by each group of patients so that we could observe the emotional distribution among different types of patients. This would allow us to better understand the emotional differences between patients with physiological and psychological diseases who participate in the forum [34].

The Boson NLP Sentiment Dictionary is built automatically from millions of microblogs, news comment sections and forums by tagging the emotion contained in the user-generated content. Negative emotion words correspond to a negative score, and positive emotion words correspond to a positive score. 

Before the sentiment analysis, we had to proceed further based on the sentiment score and content of the text. When calculating sentiment scores, if a patient posted text that contains a lot of repeated complaints and insults or recommended certain medicines (usually advertising text), the sentiment scores were extremely high or low. Thus, we examined text containing extreme emotions, which means sentiment scores greater than ± 100, and removed that which did not reflect the real emotion of patient.

**Figure 5:**
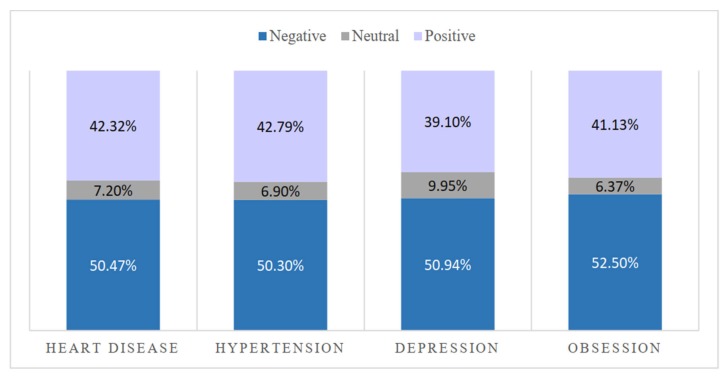
Percentage of text sentiment.

Based on the sentiment score, we plotted a percentage histogram according to the text emotion score in each OHC. Figure 5 shows the result.

As seen in Figure 5, the negative sentiment score in the posted text for each disease was approximately 50%, with the other 50% corresponding to positive and neutral sentiments. We believe that this is because both the physiological and the psychological diseases cause the patients to experience suffering, so the expressions of emotion are mostly negative. At the same time, the total of positive and neutral text was close to 50%. Thus, we can reasonably assume that whether one’s disease is psychological or physiological, the patients received social support from the community to alleviate their negative emotions.

**Figure 6:**
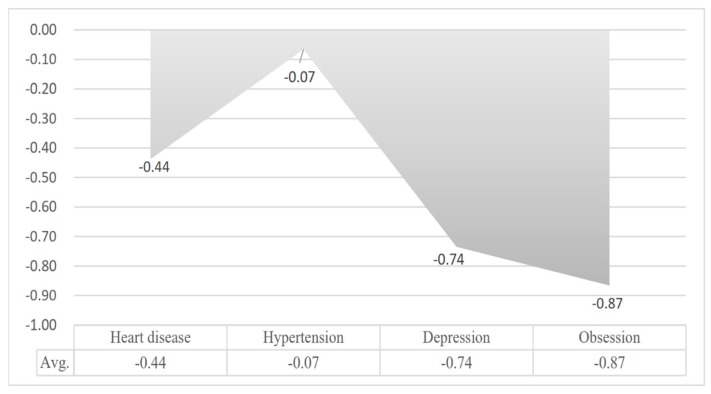
Average sentiment score.

We calculated the average emotion scores for patients based on the four diseases. As shown in Figure 6, the average sentiment scores for the four diseases were all less than 0. There was more negative than positive text among the four disease groups, which is consistent with the data presented in Figure 5. In addition, because the size of the shaded areas represents the negative sentiment scores for each of the diseases, we found that the sentiment scores of patients with heart disease and hypertension were higher than those of patients with depression and OCD; that is, the negative emotion conveyed by patients with physiological diseases was lower than that of patients with psychological diseases.

We can explain this phenomenon from the perspective of relative deprivation theory. The core idea of relative deprivation is that in comparison to a reference object, individuals or groups perceive themselves to be at a disadvantage and do not obtain the rights they deserve. Such perceptions lead to negative emotions, such as anger and grief, and psychological changes influence individual outcomes [35]. The theory is widely used in the fields of social behavior [36], social economics [37] and mental health [38].

From the perspective of relative deprivation, it can be understood that, compared to healthy persons, both patients with physiological and psychological diseases are suffering from diseases, resulting in a sense of relative deprivation. This sense reflects in the expression of negative emotions in the OHC. It is further understood that most patients with physiological diseases can cure their diseases through a specific treatment, such as surgery or medication, while most patients with psychological diseases cannot recapture their health through explicit treatments and can only recover through a combination of external treatment and self-psychological adjustment. Therefore, patients with psychological diseases experience greater difficulty obtaining health than people with physiological diseases, and the sense of relative deprivation experienced by patients with psychological diseases is greater, also the negative emotions is greater.

**Figure 7:**
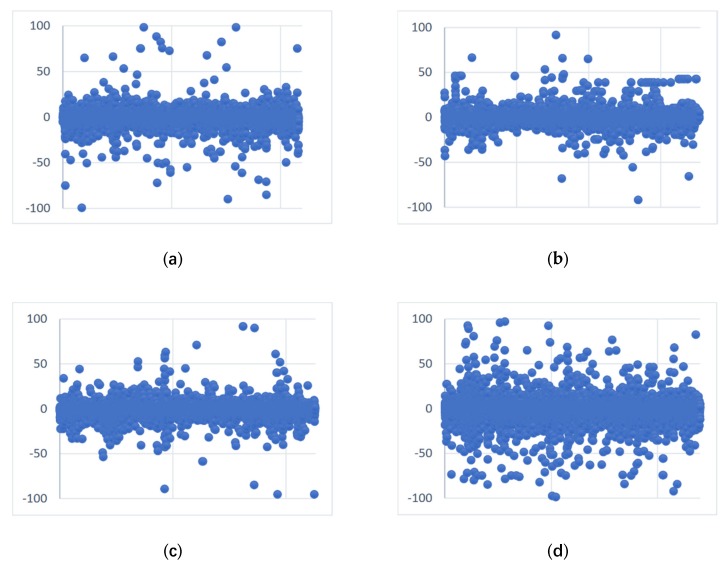
Scatter chart of sentiment score: (**a**) Distribution of sentiment score in the heart disease OHC; (**b**) distribution of sentiment score in the hypertension OHC; (**c**) distribution of sentiment score in the depression OHC; and (**d**) distribution of sentiment score in the OCD OHC.

To observe the distribution of sentiment scores among the patients according to the four diseases, we plotted scatter plots of sentiment scores based on all of the text for each disease, as shown in Figure 7a–d. The horizontal coordinates correspond to each text posted by patients, and the vertical coordinates are the sentiment scores corresponding to each of those pieces of text. We analyzed the degree of dissociation of sentiment scores in patients with heart disease, hypertension, depression and OCD.

As seen in Figure 7, the sentiment scores for each disease are evenly distributed by the 0-axis, such that the emotional scores of patients with heart disease, hypertension and depression are more concentrated, while the emotional scores of patients with OCD are more scattered. Thus, the emotional scores expressed in the text of patients with OCD are more intense and extreme. We believe that this may be because the OCD group is less concerned than other disease patients and OCD may be more difficult to cure. Thus, those patients may express emotions in the health forum that he/she cannot express in the real world, which in fact shows that OCD patients’ communication needs are not well satisfied.

## 4. Discussion

### 4.1. Principal Findings

User-generated content in online health communities comprises one of the most direct and convenient ways to learn which topics are of interest to users. In this study, we used text mining techniques to detect differences in topics of preference between patients with physiological diseases and patients with psychological diseases in the online health community. Using topic analysis and sentiment analysis, we studied user posts in four independent forums of Baidu Post Bar, discussing differences in interest and emotion demand between physiological and psychological diseases.

In contrast to previous studies, our research focused more on the differences in preference topics or emotions of patients based on the characteristics of their disease. Thus, we provided a new perspective for analyzing differences in discussion text among patients in online health communities. In addition, since we categorized patients by disease, we found similar topics as in previous studies, such as the topic of disease therapy, but we further identified particular patients who preferred to discuss this topic: patients with physiological diseases. We also found that patients with psychological diseases were more concerned about other topics, such as patients’ life environment and self-regulation. In general, we found that there were indeed differences in topics in which users were interested and in emotions between patients with physiological diseases and patients with psychological diseases in online health communities. This is reflected in the following aspects:

1. Patients with physiological diseases were more likely to discuss the difficulties and needs that they experienced in their current environment, and they were more likely to receive practical treatments. For example, patients with heart disease were more likely to discuss nondrug therapies for their disease, and those with hypertension were more likely to find drug therapies for their disease. We believe that people with physiological diseases are more concerned about how to cure their diseases. They are active in discussions on therapy recommendations in the forum, and they are also interested in sharing their own experience with others.
2. Patients with psychological diseases are more likely to describe their past experiences and moods. They are more likely to seek or provide emotional support in the community. For example, depression patients discussed their social background, interpersonal relationships, and emotional feelings, whereas OCD patients focused on self-regulation and emotional release. These two topics focused on the description of the living environment or the expression of the patients’ own feelings. We understand that patients with psychological diseases are more concerned with releasing their emotions and revealing their lives to the health community.
3. In terms of emotional performance, the mood of patients with psychological diseases was generally more negative than it was among patients with physiological diseases. This reflects that the emotional needs of patients with psychological diseases are not satisfied, especially patients with OCD. The text sentiment of OCD patients was more extreme than that of the other three diseases, which may reflect that the OCD patients receive lower social support. Therefore, their emotions are not expressed and released in daily life. As such, the OCD patients express their emotion more strongly in the anonymous online community.

The results of our study have great practical significance, which we will elaborate upon. First, from the social aspect, the construction of the national medical service system needs to listen to patients’ opinions and suggestions. Our research can help to reveal patients’ needs for medical assistance that are not being met by society. We must provide medical service tailored to different types of patients to perfect the national medical service system. Second, from the point-of-view of the online health website operator, we examined the preference topics of patients with physiological and psychological diseases. Therefore, we can construct corresponding discussion sections to improve the enthusiasm of patients in their participation in and activity on the forum. Finally, from the patients’ own perspectives, this study may help patients discover potential topics of interest, allowing them to acquire desired information or helping them more quickly during online community discussion to get online support from patients with the same problems or experiences.

### 4.2. Limitations and Future Directions

There are also some limitations to this study that need to be solved in the future. First, Baidu Post Bar is a nonprofessional online health community with a huge amount of data. Thus, the cost of data collection was low. However, because of its low entry threshold, the community lacks management. There is some noise, such as advertisements and meaningless text, in the community. In the future, researchers may try to identify specialized online communities for patients of physiological and psychological diseases to analyze their user text. Second, with the improvement or deterioration of the diseases, the topics and emotions may change. In this study, time factors could not be taken into account. In subsequent studies, time effects on patient topics and emotions may be detected by time-series analysis. Finally, the sentiment analysis adopted the existing sentiment dictionary to carry out the present analyses. As the dictionary is not completely suitable for the medical and health field, although the present analysis provided convenience, it sacrificed accuracy. Therefore, in the future, we could use machine learning combined with manual labeling methods to carry out more accurate identification of sentiment in the text of patients in the online health community.

## 5. Conclusions

This study revealed differences in topics of interest and emotions between patients with physiological and psychological diseases through Baidu Post Bar posts. Numerous researchers have shown that patients use online health communities to find treatments to cure their diseases. However, we found that the main purpose of patients with psychological diseases was to express their feelings and release their emotions rather than to seek out a specific treatment for the diseases. Additionally, patients with psychological diseases are more negative and extreme in the community, which may be caused by relatively reduced attention from the public.

## Figures and Tables

**Table 1:** Dataset.

Source	Number	Percentage
Heart disease	11,051	22.00%
Hypertension	7070	14.08%
Depression	11,558	23.01%
OCD	20,551	40.91%
Total	50,230	100.00%

**Table 2:** The topic words and topics.

Topic	Topic 1	Topic 2	Topic 3	Topic 4	Topic 5	Topic 6
Top 10 words	feel	accept	parents	surgery	meal	polypeptide
	thought	way	work	doctor	take medicine	vessel
	anxiety	cure	friends	hospital	hospital	blood
	fear	face	live	heart	cure	effect
	suffer	change	learning	inspect	doctor	diseases
	want	take in	like	children	feel	patient
	painful	solve	life	feel	exercise	body
	worry	understand	alive	color doppler ultrasound	medicine	insulin
	intention	let it be	children	after surgery	effect	natural
	doubt	make out	teacher	conditions	control	ingredient
Explanation	Patients’ feelings and thoughts	Patient self-regulation	Social environment	Nondrug therapy	Drug therapy	Professional knowledge exchange

**Table 3:** Topics and diseases examples.

Topic 1	Communities	Probability	Content
**Patients’ feelings and thoughts**	Heart disease	92.42%	I wanna sleep, I want sleep from morning to night. But I can’t, I’m gonna get up and take the pills.
	Hypertension	62.73%	After tested my blood pressure, I will hike to the mountain later, but it is just a dream.
	Depression	78.45%	I’m also moderately depressed, and I suppose it’s intermittent. Usually I just always have a headache or dizziness. I can’t move when I’m sad.
	OCD	80.16%	I feel overthought by obsessive-compulsive disorder. If I didn’t think too much, it would be painful. My friends, what am I supposed to do now?
**Topic 2**	**Communities**	**Probability**	**Content**
**Patient self-regulation**	Heart disease	67.11%	Don’t conceal it intentionally. In fact, people don’t care about you, all you worried is someone else does.
	Hypertension	81.61%	To cope with mental stress, in addition to insisting on abdominal breathing, you should also carry out mental hints. The secret is to be relax. Never imply that you are not nervous.
	Depression	92.42%	It depends on yourself, you need to be strong to face inferiority. I am not strong enough to suffer from this illness.
	OCD	83.97%	Obsessive-compulsive is very strange that your mind and consciousness will be stubborn for a long time. It is difficult for others to understand, but you should accept it freely.
**Topic 3**	**Communities**	**Probability**	**Content**
**Social environment**	Heart disease	93.42%	I am just got married and bought a house. I had economic crisis and life wasn’t easy for me.
	Hypertension	80.16%	I’m just like you used to be. You have written my thoughts. I’m happy to know you’ve gotten a job. Let’s cheer for us, everything’s getting better!
	Depression	77.27%	My parents always blame me. They used to be happy couple, but they started to blame each other and shirk their responsibilities to each other after I was born.
	OCD	82.88%	Why is OCD most occur in junior high school? I am tortured by the disease every day, and my best days of life are all destroyed. I am only 33 this year, but I feel hopeless to live.
**Topic 4**	**Communities**	**Probability**	**Content**
**Nondrug therapy**	Heart disease	78.91%	Do your surgery while you’re young to reduce risks. Don’t listen to the people who say young people do surgery will get hurt. Hurt is necessary, but young people recover faster than old people, and take me for example.
	Hypertension	76.41%	I showed the chief doctor my laboratory report, and he found that the doctor who examined me was his apprentice. What a coincidence.
	Depression	85.85%	My son had already changed his patient’s suit. I suddenly regretted bringing him here when he was examined in the hospital. I returned the money directly to take him home!
	OCD	94.79%	Do you think the 3A hospital are better? Doctors are even less responsible than those in community hospital, they hustle people through appointments.
**Topic 5**	**Communities**	**Probability**	**Content**
**Drug therapy**	Heart disease	77.27%	The doctor gave me some pills yesterday, and saying to take it for two days. If it didn’t work, I should have to ask the chief doctor for a consultation.
	Hypertension	95.69%	My pressure is 147 over 95. I plan to eat low-salt diet, do more exercise, and sleep on time, can I back to health?
	Depression	87.06%	I’d had a lot of drugs, but it didn’t work. My doctors recommend that I need to do genetic testing. Is this really useful?
	OCD	64.29%	I want to know how you are cured, and I’d like to change my pills. I’ve been OCD for two years, I’ve taken medicine but it didn’t work.
**Topic 6**	**Communities**	**Probability**	**Content**
**Professional knowledge exchange**	Heart disease	96.03%	Take two pieces of Betaloc, one ramipril, one Hydrochlorothiazide and one spironolactone table, last year my doctor added trimetazidine.
	Hypertension	93.42%	Your total cholesterol acid is very low, depending on your high-density cholesterol and low-density cholesterol.
	Depression	70.93%	Agomelatine has been heard of as an antidepressant that improves rhythm and then helps sleep without affecting the next day’s work.
	OCD	69.83%	With some glycine, the effect is obvious. Combine with gamma aminobutyric acid and magnesium sulfate, the symptoms will be improved.
